# Crystal structure of (*E*)-2,6-di-*tert*-butyl-4-{[2-(2,4-di­nitro­phen­yl)hydrazinylidene]meth­yl}phenol

**DOI:** 10.1107/S2056989016020107

**Published:** 2017-01-06

**Authors:** Md. Serajul Haque Faizi, Necmi Dege, Ashanul Haque, Valentina A. Kalibabchuk, Mustafa Cemberci

**Affiliations:** a Department of Chemistry, College of Science, Sultan Qaboos University, PO Box 36, Al-Khod 123, Muscat, Sultanate of , Oman; b Ondokuz Mayıs University, Arts and Sciences Faculty, Department of Physics, 55139 Samsun, Turkey; c Department of General Chemistry, O. O. Bohomolets National Medical University, Shevchenko Blvd. 13, 01601 Kiev, Ukraine

**Keywords:** crystal structure, hydrazine, di­nitro­benzene, di-*tert*-butyl­phenol, 3,5-di-*tert*-butyl-4-hy­droxy­benzaldehyde, 2,4-di­nitro­phenyl­hydrazine, hydrogen bonding

## Abstract

The essential part (including all the non-hydrogen atoms except two methyl carbons) of the mol­ecule of the title compound, C_21_H_26_N_4_O_5_, lies on a mirror plane, which bis­ects the *t*-butyl groups. The conformation of the C=N bond of this Schiff base compound is *E*, and there is an intra­molecular N—H⋯O hydrogen bond present, forming an *S*(6) ring motif. In the crystal, mol­ecules are linked *via* O—H⋯O hydrogen bonds, forming zigzag chains propagating along the *a-*axis direction. There are no other significant inter­molecular contacts present.

## Abstract 2

The title compound was obtained from the condensation reaction of 3,5-di-*tert*-butyl-4-hy­droxy­benzaldehyde and 2,4-di­nitro­phenyl­hydrazine. The essential part (including all the non-hydrogen atoms except two methyl carbons) of the mol­ecule lies on a mirror plane, which bis­ects the *t*-butyl groups.

## Chemical context   

Sterically hindered phenol anti-oxidants are widely used in polymers and lubricants. They can protect polymers by increasing both their process stability and their long-term stability against oxidative degradation (Yamazaki & Seguchi, 1997 ▸; Silin *et al.*, 1999 ▸). Hydrazones and Schiff bases have attracted much attention for their excellent biological properties, especially for their potential pharmacological and anti­tumor properties (Küçükgüzel *et al.*, 2006 ▸; Khattab, 2005 ▸; Karthikeyan *et al.*, 2006 ▸; Okabe *et al.*, 1993 ▸). 2,4-Di­nitro­phenyl­hydrazine is frequently used as a reagent for the characterization of aldehydes and ketones (Furniss *et al.*, 1999 ▸). Its derivatives are widely used as dyes (Guillaumont & Nakamura, 2000 ▸). They are also found to have versatile coordin­ating abilities towards different metal ions (Raj & Kurup, 2007 ▸). The present work is a part of an ongoing structural study of Schiff bases and their utilization in the synthesis of quinoxaline derivatives (Faizi *et al.*, 2016*a* ▸), fluorescence sensors (Faizi *et al.*, 2016*b* ▸) and coordination compounds (Faizi & Prisyazhnaya, 2015 ▸). We report herein on the synthesis and crystal structure of the title Schiff base compound with a sterically hindered phenol group.
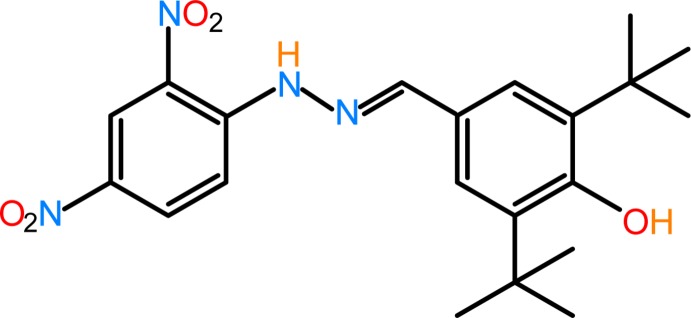


## Structural commentary   

**Figure 1:**
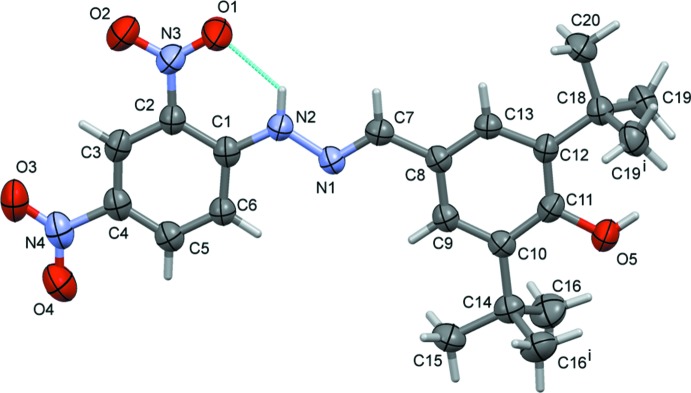
The mol­ecular structure of the title compound, with atom labelling [symmetry code: (i) *x*, −*y* + 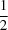, *z*]. Displacement ellipsoids are drawn at the 30% probability level. The intra­molecular N—H⋯O hydrogen bond is shown as a dashed line (see Table 1 ▸)

The mol­ecular structure of the title compound is shown in Fig. 1 ▸. All the non-hydrogen atoms except C16 and C19 lie on a crystallographic mirror plane at *y* = 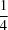: the complete *tert*-butyl groups are generated by mirror symmetry. The conformation of the C7=N1 bond of this Schiff base compound is *E*, and there is an intra­molecular N2—H2⋯O1 hydrogen bond present, forming an *S*(6) ring motif (Fig. 1 ▸ and Table 1 ▸). The N1—N2 bond length is 1.385 (6) Å and the N1=C7 bond length is 1.278 (7) Å. The bond distances and angles in the title compound are comparable to those found in a closely related structure (Fun *et al.*, 2013 ▸).

## Supra­molecular features   

**Figure 2:**
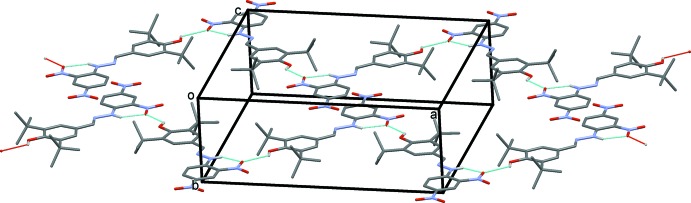
A view of the zigzag chains in the crystal structure of the title compound. The hydrogen bonds are shown as dashed lines (see Table 1 ▸). For clarity, only the H atoms involved in hydrogen bonding have been included.

In the crystal, mol­ecules are linked by O—H⋯O hydrogen bonds, forming zigzag chains propagating along the *a*-axis direction (Fig. 2 ▸ and Table 1 ▸). There are no other significant inter­molecular contacts present.

## Database survey   

There are very few examples of similar compounds in the literature. To the best of our knowledge, the recent report (Bhardwaj & Singh, 2015 ▸) of a similar compound with an hy­droxy group in the *ortho* position, capable of visual and reversible sensing of cyanide in DMSO solution, has not been characterized crystallographically. A search of the Cambridge Structural Database (CSD, Version 5.37, update May 2016; Groom *et al.*, 2016 ▸) revealed the structure of one very similar compound, *viz.* 1-(2,4-di­nitro­phen­yl)-2-[(*E*)-2,4,5-tri­meth­oxy­benzyl­idene]hydrazine (II) (Fun *et al.*, 2013 ▸), in which the 4-phenol group in the title compound is replaced by a trimeth­oxy group. In (II), the dihedral angle between the two benzene rings is 3.15 (11)°, compared to 0° in the title compound, owing to the mirror symmetry.

## Synthesis and crystallization   

A mixture of 3,5-di-*tert*-butyl-4-hy­droxy­benzaldehyde 0.100 g (0.427 mmol) and 2,4-di­nitro­phenyl­hydrazine (0.085 g, 0.427 mmol) in methanol was refluxed for 3 h in the presence of a catalytic amount of glacial acetic acid. After cooling, the red-coloured precipitate was washed with hot methanol several times, and then dried, giving a red-coloured shiny crystalline compound in high yield 170 g (96%). Yellow block-like crystals of the title compound (m.p. 372–373 K) were obtained by slow evaporation of a solution in di­chloro­methane and ethanol (5:1 *v*/*v*).

## Refinement   

Crystal data, data collection and structure refinement details are summarized in Table 2 ▸. The OH H atom was located in a difference Fourier map and refined with a distance restraint of 0.82 (2) Å with *U*_iso_(H) = 1.5*U*_eq_(O). The NH and C-bound H atoms were included in calculated positions and allowed to ride on the parent atoms: N—H = 0.86 Å, C—H = 0.93–0.96 Å with *U*_iso_(H) = 1.5*U*_eq_(C-meth­yl) and 1.2*U*_eq_(N,C) for other H atoms.

## Supplementary Material

Crystal structure: contains datablock(s) I, Global. DOI: 10.1107/S2056989016020107/su5340sup1.cif

Structure factors: contains datablock(s) I. DOI: 10.1107/S2056989016020107/su5340Isup2.hkl

CCDC reference: 1523249

Additional supporting information:  crystallographic information; 3D view; checkCIF report

## Figures and Tables

**Table 1:** Hydrogen-bond geometry (Å, °)

*D*—H⋯*A*	*D*—H	H⋯*A*	*D*⋯*A*	*D*—H⋯*A*
N2—H2⋯O1	0.86	1.96	2.583 (8)	129
O5—H5*O*⋯O1^i^	0.82 (2)	2.28 (5)	2.782 (7)	120 (4)

Symmetry code: (i) 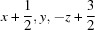.

**Table 2:** Experimental details

Crystal data	
Chemical formula	C_21_H_26_N_4_O_5_
*M*_r_	414.46
Crystal system, space group	Orthorhombic, *P* *n* *m* *a*
Temperature (K)	296
*a*, *b*, *c* (Å)	18.7651 (10), 6.9193 (4), 17.259 (1)
*V* (Å^3^)	2240.9 (2)
*Z*	4
Radiation type	Mo *K*α
μ (mm^−1^)	0.09
Crystal size (mm)	0.22 × 0.15 × 0.11

Data collection	
Diffractometer	STOE IPDS 2
Absorption correction	Integration (*X-RED32*; Stoe & Cie, 2002 ▸)
*T*_min_, *T*_max_	0.982, 0.994
No. of measured, independent and observed [*I* > 2σ(*I*)] reflections	14854, 2270, 912
*R*_int_	0.105
(sin θ/λ)_max_ (Å^−1^)	0.606

Refinement	
*R*[*F* ^2^ > 2σ(*F* ^2^)], *wR*(*F* ^2^), *S*	0.071, 0.215, 0.96
No. of reflections	2270
No. of parameters	178
No. of restraints	2
H-atom treatment	H atoms treated by a mixture of independent and constrained refinement
Δρ_max_, Δρ_min_ (e Å^−3^)	0.39, −0.16

Computer programs: *X-AREA* and *X-RED32* (Stoe & Cie, 2002 ▸), *SHELXT* (Sheldrick 2015*a* ▸), *SHELXL2016/4* (Sheldrick, 2015*b* ▸), *Mercury* (Macrae *et al.*, 2008 ▸), *WinGX* (Farrugia, 2012 ▸) and *PLATON* (Spek, 2009 ▸).

## supplementary crystallographic information

### Crystal data

**Table:**

C_21_H_26_N_4_O_5_	*D*_x_ = 1.228 Mg m^−^^3^
*M**_r_* = 414.46	Mo *K*α radiation, λ = 0.71073 Å
Orthorhombic, *P**n**m**a*	Cell parameters from 6871 reflections
*a* = 18.7651 (10) Å	θ = 1.1–26.2°
*b* = 6.9193 (4) Å	µ = 0.09 mm^−^^1^
*c* = 17.259 (1) Å	*T* = 296 K
*V* = 2240.9 (2) Å^3^	Block, yellow
*Z* = 4	0.22 × 0.15 × 0.11 mm
*F*(000) = 880	

### Data collection

**Table:**

STOE IPDS 2 diffractometer	2270 independent reflections
Radiation source: sealed X-ray tube, 12 x 0.4 mm long-fine focus	912 reflections with *I* > 2σ(*I*)
Plane graphite monochromator	*R*_int_ = 0.105
Detector resolution: 6.67 pixels mm^-1^	θ_max_ = 25.5°, θ_min_ = 1.6°
rotation method scans	*h* = −22→22
Absorption correction: integration (X-RED32; Stoe & Cie, 2002)	*k* = −8→8
*T*_min_ = 0.982, *T*_max_ = 0.994	*l* = −20→20
14854 measured reflections	

### Refinement

**Table:**

Refinement on *F*^2^	Secondary atom site location: difference Fourier map
Least-squares matrix: full	Hydrogen site location: mixed
*R*[*F*^2^ > 2σ(*F*^2^)] = 0.071	H atoms treated by a mixture of independent and constrained refinement
*wR*(*F*^2^) = 0.215	*w* = 1/[σ^2^(*F*_o_^2^) + (0.0904*P*)^2^] where *P* = (*F*_o_^2^ + 2*F*_c_^2^)/3
*S* = 0.96	(Δ/σ)_max_ < 0.001
2270 reflections	Δρ_max_ = 0.39 e Å^−^^3^
178 parameters	Δρ_min_ = −0.16 e Å^−^^3^
2 restraints	

### Special details

**Table:**

Geometry. All esds (except the esd in the dihedral angle between two l.s. planes) are estimated using the full covariance matrix. The cell esds are taken into account individually in the estimation of esds in distances, angles and torsion angles; correlations between esds in cell parameters are only used when they are defined by crystal symmetry. An approximate (isotropic) treatment of cell esds is used for estimating esds involving l.s. planes.

### Fractional atomic coordinates and isotropic or equivalent isotropic displacement parameters (Å^2^)

**Table:**

	*x*	*y*	*z*	*U*_iso_*/*U*_eq_	
O1	0.3313 (3)	0.250000	0.5101 (4)	0.172 (3)	
O2	0.2860 (3)	0.250000	0.3982 (3)	0.1219 (18)	
O3	0.4249 (4)	0.250000	0.1717 (3)	0.1208 (19)	
O4	0.5398 (4)	0.250000	0.1781 (3)	0.134 (2)	
O5	0.7461 (2)	0.250000	0.8580 (3)	0.0968 (15)	
H5O	0.738 (3)	0.250000	0.9045 (15)	0.145*	
N1	0.5301 (3)	0.250000	0.5745 (3)	0.0688 (13)	
N2	0.4674 (3)	0.250000	0.5319 (3)	0.0755 (14)	
H2	0.426908	0.250000	0.555180	0.091*	
N3	0.3381 (3)	0.250000	0.4404 (4)	0.0986 (19)	
N4	0.4801 (5)	0.250000	0.2079 (4)	0.0960 (18)	
C1	0.4705 (4)	0.250000	0.4537 (3)	0.0683 (16)	
C2	0.4077 (3)	0.250000	0.4074 (4)	0.0738 (17)	
C3	0.4119 (4)	0.250000	0.3273 (4)	0.0743 (17)	
H3	0.370779	0.250000	0.297265	0.089*	
C4	0.4780 (4)	0.250000	0.2928 (4)	0.0776 (17)	
C5	0.5396 (4)	0.250000	0.3361 (4)	0.0778 (18)	
H5A	0.583662	0.250000	0.311488	0.093*	
C6	0.5360 (3)	0.250000	0.4143 (3)	0.0707 (16)	
H6	0.578077	0.250000	0.442879	0.085*	
C7	0.5207 (3)	0.250000	0.6478 (4)	0.0686 (16)	
H7	0.474375	0.250000	0.666931	0.082*	
C8	0.5798 (3)	0.250000	0.7027 (3)	0.0642 (15)	
C9	0.6498 (3)	0.250000	0.6776 (3)	0.0677 (15)	
H9	0.659076	0.250000	0.624701	0.081*	
C10	0.7066 (3)	0.250000	0.7293 (3)	0.0666 (16)	
C11	0.6887 (3)	0.250000	0.8089 (4)	0.0739 (17)	
C12	0.6187 (3)	0.250000	0.8373 (3)	0.0663 (15)	
C13	0.5656 (3)	0.250000	0.7812 (3)	0.0656 (15)	
H13	0.518293	0.250000	0.797276	0.079*	
C14	0.7841 (3)	0.250000	0.7007 (4)	0.0775 (18)	
C15	0.7881 (4)	0.250000	0.6117 (4)	0.098 (2)	
H15A	0.764903	0.136762	0.592106	0.146*	
H15B	0.837191	0.250000	0.596024	0.146*	
C16	0.8233 (2)	0.4334 (8)	0.7297 (3)	0.1114 (18)	
H16A	0.797329	0.546218	0.713820	0.167*	
H16B	0.870376	0.437202	0.708115	0.167*	
H16C	0.826331	0.430459	0.785250	0.167*	
C18	0.6013 (3)	0.250000	0.9242 (3)	0.0755 (18)	
C19	0.6312 (3)	0.4323 (7)	0.9640 (2)	0.0973 (16)	
H19A	0.679150	0.408097	0.980849	0.146*	
H19B	0.602137	0.464417	1.007856	0.146*	
H19C	0.631039	0.537943	0.927893	0.146*	
C20	0.5207 (4)	0.250000	0.9375 (4)	0.102 (2)	
H20A	0.500334	0.363387	0.914288	0.154*	
H20B	0.511112	0.250000	0.991999	0.154*	

### Atomic displacement parameters (Å^2^)

**Table:**

	*U*^11^	*U*^22^	*U*^33^	*U*^12^	*U*^13^	*U*^23^
O1	0.081 (4)	0.362 (10)	0.073 (4)	0.000	0.007 (3)	0.000
O2	0.083 (4)	0.164 (5)	0.118 (4)	0.000	−0.024 (3)	0.000
O3	0.147 (5)	0.135 (5)	0.080 (4)	0.000	−0.034 (4)	0.000
O4	0.147 (5)	0.177 (6)	0.078 (4)	0.000	0.013 (4)	0.000
O5	0.077 (3)	0.136 (4)	0.078 (3)	0.000	−0.017 (3)	0.000
N1	0.072 (3)	0.078 (3)	0.056 (3)	0.000	0.000 (3)	0.000
N2	0.063 (3)	0.103 (4)	0.061 (3)	0.000	−0.005 (3)	0.000
N3	0.068 (4)	0.145 (6)	0.083 (4)	0.000	−0.009 (4)	0.000
N4	0.130 (6)	0.091 (4)	0.068 (4)	0.000	−0.009 (4)	0.000
C1	0.083 (4)	0.059 (4)	0.064 (4)	0.000	−0.008 (4)	0.000
C2	0.078 (5)	0.081 (4)	0.063 (4)	0.000	−0.010 (4)	0.000
C3	0.082 (5)	0.063 (4)	0.078 (5)	0.000	−0.022 (4)	0.000
C4	0.102 (5)	0.066 (4)	0.065 (4)	0.000	−0.007 (4)	0.000
C5	0.086 (5)	0.077 (4)	0.070 (4)	0.000	0.001 (4)	0.000
C6	0.072 (4)	0.079 (4)	0.061 (4)	0.000	−0.004 (3)	0.000
C7	0.067 (4)	0.069 (4)	0.069 (4)	0.000	0.003 (4)	0.000
C8	0.069 (4)	0.066 (4)	0.058 (4)	0.000	0.001 (3)	0.000
C9	0.077 (4)	0.068 (4)	0.058 (3)	0.000	−0.001 (4)	0.000
C10	0.069 (4)	0.068 (4)	0.063 (4)	0.000	0.003 (3)	0.000
C11	0.064 (4)	0.080 (4)	0.078 (4)	0.000	−0.016 (4)	0.000
C12	0.069 (4)	0.070 (4)	0.060 (4)	0.000	−0.011 (3)	0.000
C13	0.061 (4)	0.072 (4)	0.064 (4)	0.000	0.002 (3)	0.000
C14	0.075 (4)	0.076 (4)	0.082 (4)	0.000	0.001 (4)	0.000
C15	0.084 (5)	0.125 (6)	0.084 (5)	0.000	0.019 (4)	0.000
C16	0.086 (3)	0.122 (4)	0.126 (5)	−0.028 (3)	0.000 (3)	0.000 (4)
C18	0.076 (4)	0.090 (5)	0.061 (4)	0.000	−0.004 (3)	0.000
C19	0.115 (4)	0.109 (4)	0.068 (3)	−0.002 (3)	−0.002 (3)	−0.013 (3)
C20	0.089 (5)	0.147 (7)	0.071 (4)	0.000	0.017 (4)	0.000

### Geometric parameters (Å, º)

**Table:**

O1—N3	1.209 (7)	C9—H9	0.9300
O2—N3	1.219 (7)	C10—C11	1.415 (8)
O3—N4	1.210 (7)	C10—C14	1.536 (8)
O4—N4	1.232 (8)	C11—C12	1.401 (8)
O5—C11	1.370 (7)	C12—C13	1.390 (8)
O5—H5O	0.816 (19)	C12—C18	1.536 (8)
N1—C7	1.278 (7)	C13—H13	0.9300
N1—N2	1.385 (6)	C14—C15	1.538 (9)
N2—C1	1.352 (7)	C14—C16	1.549 (6)
N2—H2	0.8600	C14—C16^i^	1.549 (6)
N3—C2	1.424 (8)	C15—H15A	0.96
N4—C4	1.466 (8)	C15—H15B	0.96
C1—C6	1.405 (8)	C15—H15A^i^	0.96
C1—C2	1.422 (8)	C16—H16A	0.9600
C2—C3	1.385 (8)	C16—H16B	0.9600
C3—C4	1.374 (8)	C16—H16C	0.9600
C3—H3	0.9300	C18—C20	1.530 (9)
C4—C5	1.376 (9)	C18—C19	1.542 (5)
C5—C6	1.352 (8)	C18—C19^i^	1.542 (5)
C5—H5A	0.9300	C19—H19A	0.9600
C6—H6	0.9300	C19—H19B	0.9600
C7—C8	1.458 (8)	C19—H19C	0.9600
C7—H7	0.9300	C20—H20A	0.96
C8—C13	1.380 (7)	C20—H20B	0.96
C8—C9	1.383 (8)	C20—H20A^i^	0.96
C9—C10	1.390 (8)		
			
C11—O5—H5O	118 (4)	C12—C11—C10	124.2 (6)
C7—N1—N2	114.1 (5)	C13—C12—C11	115.4 (5)
C1—N2—N1	119.6 (5)	C13—C12—C18	121.9 (5)
C1—N2—H2	120.2	C11—C12—C18	122.7 (5)
N1—N2—H2	120.2	C8—C13—C12	123.0 (6)
O1—N3—O2	120.6 (6)	C8—C13—H13	118.5
O1—N3—C2	119.7 (6)	C12—C13—H13	118.5
O2—N3—C2	119.7 (6)	C10—C14—C15	111.5 (5)
O3—N4—O4	124.2 (7)	C10—C14—C16	110.2 (3)
O3—N4—C4	119.5 (8)	C15—C14—C16	107.4 (4)
O4—N4—C4	116.3 (7)	C10—C14—C16^i^	110.2 (3)
N2—C1—C6	121.3 (6)	C15—C14—C16^i^	107.4 (4)
N2—C1—C2	121.8 (6)	C16—C14—C16^i^	110.0 (6)
C6—C1—C2	117.0 (5)	C14—C15—H15A	109.3
C3—C2—C1	120.9 (6)	C14—C15—H15B	109.2
C3—C2—N3	116.9 (6)	H15A—C15—H15B	109.6
C1—C2—N3	122.3 (6)	C14—C15—H15A^i^	109.3 (4)
C4—C3—C2	118.9 (6)	H15A—C15—H15A^i^	109.7
C4—C3—H3	120.5	H15B—C15—H15A^i^	109.6
C2—C3—H3	120.5	C14—C16—H16A	109.5
C3—C4—C5	121.5 (6)	C14—C16—H16B	109.5
C3—C4—N4	117.2 (7)	H16A—C16—H16B	109.5
C5—C4—N4	121.3 (7)	C14—C16—H16C	109.5
C6—C5—C4	120.1 (7)	H16A—C16—H16C	109.5
C6—C5—H5A	120.0	H16B—C16—H16C	109.5
C4—C5—H5A	120.0	C20—C18—C12	110.9 (5)
C5—C6—C1	121.7 (6)	C20—C18—C19	107.1 (4)
C5—C6—H6	119.2	C12—C18—C19	110.9 (3)
C1—C6—H6	119.2	C20—C18—C19^i^	107.1 (4)
N1—C7—C8	122.6 (6)	C12—C18—C19^i^	110.9 (3)
N1—C7—H7	118.7	C19—C18—C19^i^	109.8 (5)
C8—C7—H7	118.7	C18—C19—H19A	109.5
C13—C8—C9	119.4 (6)	C18—C19—H19B	109.5
C13—C8—C7	119.4 (6)	H19A—C19—H19B	109.5
C9—C8—C7	121.2 (5)	C18—C19—H19C	109.5
C8—C9—C10	121.8 (6)	H19A—C19—H19C	109.5
C8—C9—H9	119.1	H19B—C19—H19C	109.5
C10—C9—H9	119.1	C18—C20—H20A	109.3
C9—C10—C11	116.2 (6)	C18—C20—H20B	109.5
C9—C10—C14	121.4 (5)	H20A—C20—H20B	109.6
C11—C10—C14	122.4 (5)	C18—C20—H20A^i^	109.3 (5)
O5—C11—C12	121.4 (6)	H20A—C20—H20A^i^	109.6
O5—C11—C10	114.4 (6)	H20B—C20—H20A^i^	109.6
			
C7—N1—N2—C1	180.000 (1)	C7—C8—C9—C10	180.000 (1)
N1—N2—C1—C6	0.000 (1)	C8—C9—C10—C11	0.000 (2)
N1—N2—C1—C2	180.000 (1)	C8—C9—C10—C14	180.000 (1)
N2—C1—C2—C3	180.000 (1)	C9—C10—C11—O5	180.000 (1)
C6—C1—C2—C3	0.000 (1)	C14—C10—C11—O5	0.000 (1)
N2—C1—C2—N3	0.000 (1)	C9—C10—C11—C12	0.000 (2)
C6—C1—C2—N3	180.000 (1)	C14—C10—C11—C12	180.000 (1)
O1—N3—C2—C3	180.000 (1)	O5—C11—C12—C13	180.000 (1)
O2—N3—C2—C3	0.000 (1)	C10—C11—C12—C13	0.000 (2)
O1—N3—C2—C1	0.000 (1)	O5—C11—C12—C18	0.000 (2)
O2—N3—C2—C1	180.000 (1)	C10—C11—C12—C18	180.000 (2)
C1—C2—C3—C4	0.000 (1)	C9—C8—C13—C12	0.000 (2)
N3—C2—C3—C4	180.000 (1)	C7—C8—C13—C12	180.000 (1)
C2—C3—C4—C5	0.000 (1)	C11—C12—C13—C8	0.000 (2)
C2—C3—C4—N4	180.000 (1)	C18—C12—C13—C8	180.000 (2)
O3—N4—C4—C3	0.000 (1)	C9—C10—C14—C15	0.000 (2)
O4—N4—C4—C3	180.000 (1)	C11—C10—C14—C15	180.000 (1)
O3—N4—C4—C5	180.000 (1)	C9—C10—C14—C16	119.2 (4)
O4—N4—C4—C5	0.000 (1)	C11—C10—C14—C16	−60.8 (4)
C3—C4—C5—C6	0.000 (1)	C9—C10—C14—C16^i^	−119.2 (4)
N4—C4—C5—C6	180.000 (1)	C11—C10—C14—C16^i^	60.8 (4)
C4—C5—C6—C1	0.000 (1)	C13—C12—C18—C20	0.000 (2)
N2—C1—C6—C5	180.000 (1)	C11—C12—C18—C20	180.000 (2)
C2—C1—C6—C5	0.000 (1)	C13—C12—C18—C19	−118.8 (4)
N2—N1—C7—C8	180.000 (1)	C11—C12—C18—C19	61.2 (4)
N1—C7—C8—C13	180.000 (1)	C13—C12—C18—C19^i^	118.8 (4)
N1—C7—C8—C9	0.000 (2)	C11—C12—C18—C19^i^	−61.2 (4)
C13—C8—C9—C10	0.000 (2)		

Symmetry code: (i) *x*, −*y*+1/2, *z*.

### Hydrogen-bond geometry (Å, º)

**Table:**

*D*—H···*A*	*D*—H	H···*A*	*D*···*A*	*D*—H···*A*
N2—H2···O1	0.86	1.96	2.583 (8)	129
O5—H5*O*···O1^ii^	0.82 (2)	2.28 (5)	2.782 (7)	120 (4)

Symmetry code: (ii) *x*+1/2, *y*, −*z*+3/2.

## References

[bb1] Bhardwaj, S. & Singh, A. K. (2015). *J. Hazard. Mater.* **296**, 54–60. 10.1016/j.jhazmat.2015.04.043 25913671

[bb2] Faizi, M. S. H., Ali, A. & Potaskalov, V. A. (2016*a*). *Acta Cryst.* E**72**, 1366–1369. 10.1107/S205698901601344X PMC5050754 27746919

[bb3] Faizi, M. S. H., Gupta, S., Mohan, V. K., Jain, K. V. & Sen, P. (2016*b*). *Sens. Actuators B Chem.* **222**, 15–20.

[bb4] Faizi, M. S. H. & Prisyazhnaya, E. V. (2015). *Acta Cryst.* E**71**, m175–m176. 10.1107/S2056989015015790 PMC4555402 26396873

[bb5] Farrugia, L. J. (2012). *J. Appl. Cryst.* **45**, 849–854.

[bb6] Fun, H.-K., Chantrapromma, S., Nilwanna, B., Kobkeatthawin, T. & Boonnak, N. (2013). *Acta Cryst.* E**69**, o1203–o1204. 10.1107/S1600536813018345 PMC3793714 24109301

[bb7] Furniss, B. S., Hannaford, A. J., Smith, P. W. G. & Tatchell, A. R. (1999). *Vogel’s Textbook of Practical Organic Chemistry*, 5th ed. London: Longmans.

[bb8] Groom, C. R., Bruno, I. J., Lightfoot, M. P. & Ward, S. C. (2016). *Acta Cryst.* B**72**, 171–179. 10.1107/S2052520616003954 PMC4822653 27048719

[bb9] Guillaumont, D. & Nakamura, S. (2000). *Dyes Pigments*, **46**, 85–92.

[bb10] Karthikeyan, M. S., Prasad, D. J., Poojary, B., Bhat, K. S., Holla, B. S. & Kumari, N. S. (2006). *Bioorg. Med. Chem.* **14**, 7482–7489. 10.1016/j.bmc.2006.07.015 16879972

[bb11] Khattab, S. N. (2005). *Molecules*, **10**, 1218–1228. 10.3390/10091218 PMC6147684 18007388

[bb12] Küçükgüzel, G., Kocatepe, A., De Clercq, E., Şahin, F. & Güllüce, M. (2006). *Eur. J. Med. Chem.* **41**, 353–359. 10.1016/j.ejmech.2005.11.005 16414150

[bb13] Macrae, C. F., Bruno, I. J., Chisholm, J. A., Edgington, P. R., McCabe, P., Pidcock, E., Rodriguez-Monge, L., Taylor, R., van de Streek, J. & Wood, P. A. (2008). *J. Appl. Cryst.* **41**, 466–470.

[bb14] Okabe, N., Nakamura, T. & Fukuda, H. (1993). *Acta Cryst.* C**49**, 1678–1680.

[bb15] Raj, B. N. B. & Kurup, M. R. P. (2007). *Spectrochim. Acta Part A*, **66**, 898–903. 10.1016/j.saa.2006.05.006 17045519

[bb16] Sheldrick, G. M. (2015*a*). *Acta Cryst.* A**71**, 3–8.

[bb17] Sheldrick, G. M. (2015*b*). *Acta Cryst.* C**71**, 3–8.

[bb18] Silin, M. A., Kelaren, V. I., Abu-Ammar, V., Putkaradze, D. Kh. & Golubeva, I. A. (1999). *Pet. Chem.* **40**, 209–214.

[bb19] Spek, A. L. (2009). *Acta Cryst.* D**65**, 148–155. 10.1107/S090744490804362X PMC2631630 19171970

[bb20] Stoe & Cie (2002). *X-AREA* and *X-RED32*. Stoe & Cie, Darmstadt, Germany.

[bb21] Yamazaki, T. & Seguchi, T. (1997). *J. Polym. Sci. A Polym. Chem.* **35**, 2431–2439.

